# Ceiling effects in the EOSQ-24 may limit its ability to assess treatment outcomes in early-onset scoliosis

**DOI:** 10.1007/s43390-026-01293-2

**Published:** 2026-01-31

**Authors:** Antti Juhani Saarinen

**Affiliations:** Department of Orthopedics and Traumatology, Turku University Hospital and University of Turku, Luolavuorentie 2, 20700 Turku, Finland

**Keywords:** Patient reported outcome measure, Methodology, Quality of life, Early onset scoliosis

## Abstract

**Background:**

The 24-item Early-Onset Scoliosis Questionnaire (EOSQ-24) is a condition-specific instrument to assess health-related quality of life in children with early-onset scoliosis (EOS). Previous studies have raised concerns regarding ceiling effects, i.e. clustering of scores at the upper limit, which may reduce the ability of the EOSQ-24 to detect clinical improvements.

**Methods:**

A structured review of published clinical studies reporting EOSQ-24 scores was performed. Data were standardized to a 0–100 scale. For each domain weighted mean scores, standard deviations (SD), and ceiling effect percentages were calculated. A ceiling effect ≥ 15% was considered significant.

**Results:**

Data from 16 studies were included. Significant ceiling effects were observed in the following domains: pulmonary function (28%), physical function (25%), overall satisfaction (22%), transfer (20%), financial burden (22%), daily living (18%), fatigue/energy level (18%), and emotion (16%). Lower ceiling effects were noted in general health, pain/discomfort, parental burden, and child/parent satisfaction domains.

**Conclusions:**

Eight of EOSQ-24 domains exhibit marked ceiling effects, potentially limiting responsiveness to treatment effects in patients with high baseline function. These findings support consideration of revised scoring strategies or adjunctive measures in EOS outcome assessment. The presence of marked ceiling effect should be noted as a limitation in studies. Objective pulmonary function testing should be implemented in future studies.

**Supplementary Information:**

The online version contains supplementary material available at 10.1007/s43390-026-01293-2.

## Introduction

The 24-item Early-Onset Scoliosis Questionnaire (EOSQ-24) is a condition-specific instrument to assess health-related quality of life in children with early-onset scoliosis (EOS) [1]. The EOSQ-24 can be completed by either the patient or their caregiver. The EOSQ-24 consists of 24 items across ten domains: General Health, Pain/Discomfort, Pulmonary Function, Transfer, Physical Function, Daily Living, Fatigue, Emotion, Child Satisfaction, and Parent Satisfaction. EOS can be divided into syndromic, neuromuscular, congenital, and idiopathic scoliosis [2]. Etiology is significantly associated with the EOSQ-24 scores in patients with EOS, with children with syndromic or neuromuscular scoliosis reporting lower scores than patients with congenital or idiopathic scoliosis [3].

A ceiling effect occurs when a substantial proportion of respondents attain the highest possible score on a scale, which compresses variability at the top end and reduces sensitivity to detect further improvement [4]. In patient reported outcome measure (PROM) methodology, thresholds of 15% of respondents at the maximum are commonly used to flag a problematic ceiling effect and potential limitations in content validity and responsiveness [4].

While the EOSQ-24 has demonstrated good psychometric properties, concerns have been raised regarding ceiling effects, i.e. clustering of scores at the upper limit, which reduce sensitivity to detect change [5–10]. Ceiling effects arise when item difficulty is poorly matched to respondent ability, resulting in score clustering at the upper bound. Several EOSQ-24 domains assess overt functional limitation or severe symptom states (e.g., respiratory compromise, need for assistance with transfers), which may be absent in many treated or higher-functioning patients. When combined with a limited five-level response structure and proxy-reported assessments, this design may insufficiently discriminate among patients with mild residual impairment, predisposing several domains to ceiling effects.

The aim of this study was to systematically review clinical studies using the EOSQ-24 and quantify domain-specific ceiling effects across published EOS populations.

## Methods

A systematic review was performed for clinical studies reporting EOSQ-24 outcomes in early-onset scoliosis. PubMed/MEDLINE, Embase, and Scopus databases were searched for ‘“early-onset scoliosis questionnaire” OR “EOSQ-24”’. Preliminary screening was performed using titles and abstracts. The inclusion criteria were clinical research setting using EOSQ-24 as an outcome. EOSQ-24 validation studies were excluded, as these patient samples may not reflect relevant clinical settings. Full texts were screened for final inclusion. If stated, the primary outcome timepoint EOSQ-24 was collected. If not specified, the most recent follow-up was considered the primary outcome for this review. When multiple groups were reported within a study, each group was treated as a separate data point. Studies reporting only subsets of EOSQ-24 items were also considered eligible. The original EOSQ-24 included two items in the Satisfaction domain: Child Satisfaction and Parent Satisfaction. However, some studies reported only a single overall satisfaction score; in such cases, the data was collected in Overall Satisfaction and were not included in Parent or Child Satisfaction domains. Each EOSQ-24 item is scored on a five-point Likert scale. Domain scores are calculated as the mean of item responses within that domain and expressed on a scale with a maximum score of 100. When EOSQ-24 domain scores were reported using score ranges other than the conventional 0–100 scale, they were linearly re-expressed on the 0–100 scale to allow comparison across studies. Weighted means for each domain were calculated using sample sizes.

For each EOSQ-24 domain, reported mean, standard deviation (SD), sample size, and scale range were extracted. When only median and interquartile range (IQR) were available, mean and SD were approximated using the method by Wan et al., where the mean is set equal to the median and SD estimated as IQR/1.35 [11]. When normal distribution is assumed, the proportion of the sample expected to reach or exceed the maximum possible score (100 points) can be estimated as 1 − Φ((100 − mean)/SD), where Φ denotes the cumulative distribution function of the standard normal distribution [12]. Ceiling proportions were then summarized across domains. Ceiling effect of at least 15% was considered as significant. Because EOSQ-24 domain scores are ordinal, bounded, and frequently right-skewed, the assumption of normality may underestimate the true proportion of observations at the maximum score. Consequently, calculated ceiling proportions should be interpreted as conservative estimates rather than exact values.

## Results

**Fig. 1:**
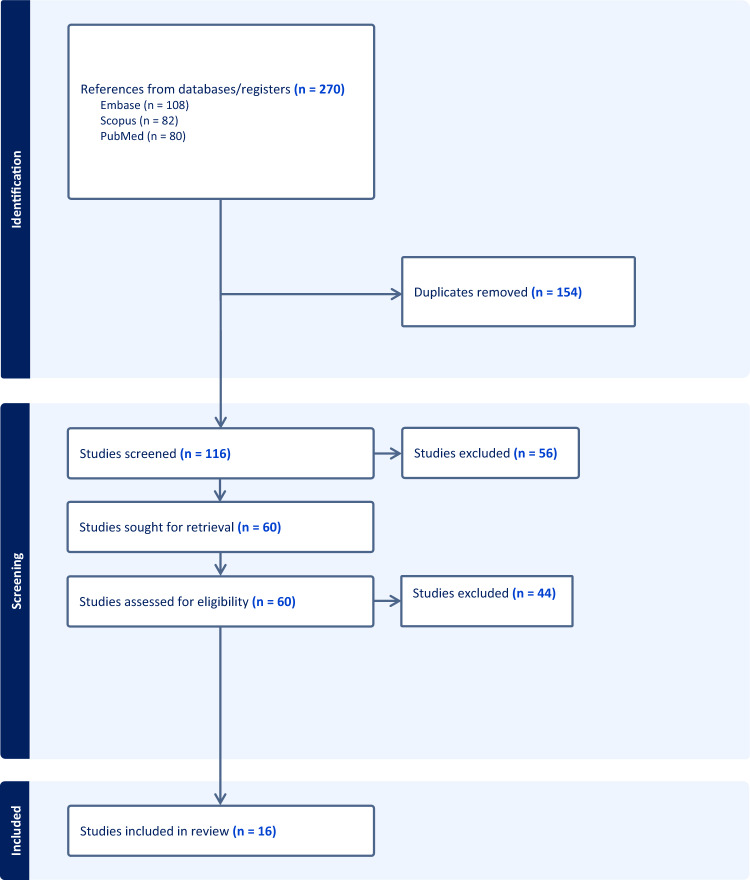
Flowchart of the study selection process

In all, 116 references were found (Fig. 1). After the preliminary screening, 60 studies were included for the full text review. Studies that lacked SD or IQR were excluded. Two studies reported adjusted EOSQ-24 data without further explanation and were excluded from the analysis. In total, 16 studies comprising 32 groups and 357 domains met the inclusion criteria (Supplementary data) [3, 13–25]. Five studies reported medians and IQRs.

Every included study had at least one EOSQ-24 domain with a significant ceiling effect present. Pulmonary Function, Transfer, and Financial Burden items had the largest incidence of ceiling effect, and General Health, Parental Burden, and Child Satisfaction the lowest (Table 1).

**Table 1:** Weighted mean scores and mean ceiling effects for each EOSQ-24 item

Item	Mean score	SD	Mean ceiling effect	SD	Percentage of study groups with ceiling effect minimum of 15%
General health	74.1	5.8	7.7	5.0	16
Pain/discomfort	73.8	8.0	13.0	7.8	38
Pulmonary function	88.3	7.3	28.1	11.8	97
Transfer	79.6	12.7	20.4	13.6	79
Physical function	76.6	16.5	24.7	14.8	71
Daily living	66.3	21.6	17.5	15.1	55
Fatigue and energy level	74.0	10.5	17.9	11.3	55
Emotion	76.7	8.4	15.9	7.9	44
Parental burden	71.6	10.8	9.4	9.7	17
Financial burden	78.3	10.8	22.1	11.4	77
Child satisfaction	70.1	8.1	12.1	7.6	25
Parental satisfaction	71.0	8.1	13.7	7.7	42
Overall satisfaction	69.5	28.8	21.9	22.1	33

When weighted means were calculated for all studies, a mean ceiling effect of at least 15% was observed in eight of the 11 analyzed domains (including Overall Satisfaction analyzed separately from Parent and Child Satisfaction**)**: Pulmonary Function (28%), Physical Function (25%), Overall Satisfaction (22%), Transfer (20%), Financial Burden (22%), Daily Living (18%), Fatigue/Energy Level (18%), and Emotion (16%) (Table 1). In terms of frequency, marked proportion of the study groups showed ceiling effects ≥ 15% in Pulmonary Function (97% of the groups), Transfer (79% of the groups), Financial Burden (77% of the groups), and Physical Function (71% of the groups), whereas General Health (16% of the groups) and Parental Burden (17% of the groups) were least affected (Table 1).

## Discussion

In this review, the EOSQ-24 demonstrated substantial ceiling effects across multiple domains. Ceiling effects of ≥ 15% were identified in eight of the domains: Pulmonary Function, Transfer, Physical Function, Daily Living, Fatigue/Energy Level, Emotion, Financial Burden, and Overall Satisfaction. Domains such as Pain/Discomfort and General Health demonstrated lower ceiling effects. These domains include items assessing subjective symptom intensity and overall health perception across a broader severity range, which may improve person–item targeting and preserve variability at higher functioning levels. These results indicate that ceiling effects are widespread in the EOSQ-24 and may limit its ability to detect treatment-related improvements, particularly in higher-functioning patients. Notably, Pulmonary Function, Transfer, Financial Burden, and Physical Function exceeded the 15% threshold in most study groups, underscoring the consistency of these findings across studies.

The Pulmonary Function domain demonstrated the most pronounced ceiling effect, with nearly all study groups exceeding the 15% threshold. Items within this domain primarily assess overt respiratory compromise rather than exertional limitation, nocturnal symptoms, or early functional decline. As a result, many patients without advanced pulmonary impairment may cluster at the maximum score despite clinically meaningful differences in respiratory status. This ceiling clustering likely reflects item content rather than absence of pulmonary morbidity and limits the domain’s sensitivity in higher-functioning patients. The EOSQ-24 Pulmonary Domain may therefore be most informative in patients with severe neuromuscular or syndromic EOS, while offering limited discrimination in idiopathic or moderately affected cohorts. Several previous studies have noted the marked ceiling effect in the Pulmonary Function domain [5, 8–10]. Clinical pulmonary function testing should be implemented to adequately evaluate pulmonary function related outcomes in patients with EOS [26].

The presence of ceiling effects beyond the commonly accepted 15% threshold raises concerns about the responsiveness of the EOSQ-24. Once patients score near the maximum, further improvements cannot be captured, potentially underestimating treatment benefits. Moreover, when marked ceiling effects are present, comparisons between groups may become unreliable, as differences in outcomes cannot be fully expressed. When baseline scores approach the maximum, even clinically meaningful improvement cannot be captured, reducing standardized response means and effect sizes and increasing the risk of false-negative conclusions. In clinical decision making, this may obscure treatment benefits, particularly when comparing surgical techniques or longitudinal outcomes in higher-functioning patients. These limitations are consistent with those described for other PROMs and highlight the need to complement EOSQ-24 with objective measures to ensure adequate assessment across the full spectrum of disease severity.

This review has limitations. In some studies, mean and SD were approximated from medians and IQRs, which may have introduced imprecision; however, the principal conclusions of this study remain unaffected. Heterogeneity in study populations and designs may also have contributed to variability. The ≥ 15% threshold was adopted a priori based on widely used PROM quality criteria rather than derived empirically. Proxy reporting may contribute to ceiling effects, as caregivers may be less sensitive to subtle functional or symptomatic changes, particularly in higher-functioning children. Respondent type was inconsistently reported and rarely stratified across studies, precluding quantitative comparison of ceiling effects by respondent. Some studies reported aggregated satisfaction scores that likely represent mixed constructs. Although analyzed separately, this heterogeneity may obscure domain-specific ceiling behavior. The reliance on distribution-based estimation rather than individual-level data introduces uncertainty, particularly given the bounded and non-normal nature of EOSQ-24 scores. However, the near-universal presence of marked ceiling effects across studies and domains suggests that these findings are not solely attributable to distributional assumptions. Ceiling effects are likely influenced by patient age, etiology, disease severity, treatment stage, and respondent type. Although the included studies encompassed a wide spectrum of EOS populations, subgroup-specific analyses were not feasible due to inconsistent reporting of stratified EOSQ-24 summary statistics. Future studies using individual-level data are needed to distinguish ceiling effects driven by case-mix characteristics from those reflecting inherent limitations of item content. Nonetheless, the consistent presence of ceiling effects across studies suggests the presence of ceiling effects is an inherent characteristic of the EOSQ-24 rather than a study-specific phenomenon.

## Conclusions

Ceiling effects are common in the EOSQ-24, particularly in functional and pulmonary items, and may limit its ability to capture treatment-related change. The presence of ceiling effects should be reported and recognized as a limitation in clinical studies. In practice, reliance on EOSQ-24 alone may underestimate treatment benefits in higher-functioning patients. Complementary use of objective measures, particularly pulmonary function testing, is recommended in future studies. When PROMs are used as primary outcomes, awareness of domain-specific ceiling effects is essential for interpretation.

## Supplementary Information

Below is the link to the electronic supplementary material.

Supplementary file1 (XLSX 18 KB)

## Data Availability

Data is available for a reasonable request from the corresponding author.
